# Neurofilament light: a possible prognostic biomarker for treatment of vascular contributions to cognitive impairment and dementia

**DOI:** 10.1186/s12974-021-02281-1

**Published:** 2021-10-15

**Authors:** Christina Hoyer-Kimura, John P. Konhilas, Heidi M. Mansour, Robin Polt, Kristian P. Doyle, Dean Billheimer, Meredith Hay

**Affiliations:** 1 Department of Physiology, The University of Arizona, Tucson, AZ USA; 2 Department of Nutritional Sciences, The University of Arizona, Tucson, AZ USA; 3 Department of Biomedical Engineering, The University of Arizona, Tucson, AZ USA; 4 Sarver Molecular Cardiovascular Research Program, The University of Arizona, Tucson, AZ USA; 5 Department of Pharmacy, Skaggs Pharmaceutical Sciences Center, The University of Arizona, Tucson, AZ USA; 6 Department of Medicine, Division of Translational and Regenerative Medicine, The University of Arizona, Tucson, AZ USA; 7 Department of Chemistry and Biochemistry, The University of Arizona, Tucson, AZ USA; 8 Department of Immunobiology, The University of Arizona, Tucson, AZ USA; 9 Department of Neurology, The University of Arizona, Tucson, AZ USA; 10 Evelyn F. McKnight Brain Institute, The University of Arizona, Tucson, AZ USA; 11 Department of Epidemiology and Biostatistics, The University of Arizona, Tucson, AZ USA; 12 ProNeurogen, Inc, The University of Arizona, Tucson, AZ USA

**Keywords:** Neurofilament light (NfL), Biomarker, Vascular contributions to cognitive impairment and dementia, Inflammation, PNA5, Angiotensin-(1–7)

## Abstract

**Background:**

Decreased cerebral blood flow and systemic inflammation during heart failure (HF) increase the risk for vascular contributions to cognitive impairment and dementia (VCID) and Alzheimer disease-related dementias (ADRD). We previously demonstrated that PNA5, a novel glycosylated angiotensin 1–7 (Ang-(1–7)) Mas receptor (MasR) agonist peptide, is an effective therapy to rescue cognitive impairment in our preclinical model of VCID. Neurofilament light (NfL) protein concentration is correlated with cognitive impairment and elevated in neurodegenerative diseases, hypoxic brain injury, and cardiac disease. The goal of the present study was to determine (1) if treatment with Ang-(1–7)/MasR agonists can rescue cognitive impairment and decrease VCID-induced increases in NfL levels as compared to HF-saline treated mice and, (2) if NfL levels correlate with measures of cognitive function and brain cytokines in our VCID model.

**Methods:**

VCID was induced in C57BL/6 male mice via myocardial infarction (MI). At 5 weeks post-MI, mice were treated with daily subcutaneous injections for 24 days, 5 weeks after MI, with PNA5 or angiotensin 1–7 (500 microg/kg/day or 50 microg/kg/day) or saline (*n* = 15/group). Following the 24-day treatment protocol, cognitive function was assessed using the Novel Object Recognition (NOR) test. Cardiac function was measured by echocardiography and plasma concentrations of NfL were quantified using a Quanterix Simoa assay. Brain and circulating cytokine levels were determined with a MILLIPLEX MAP Mouse High Sensitivity Multiplex Immunoassay. Treatment groups were compared via ANOVA, significance was set at *p* < 0.05.

**Results:**

Treatment with Ang-(1–7)/MasR agonists reversed VCID-induced cognitive impairment and significantly decreased NfL levels in our mouse model of VCID as compared to HF-saline treated mice. Further, NfL levels were significantly negatively correlated with cognitive scores and the concentrations of multiple pleiotropic cytokines in the brain.

**Conclusions:**

These data show that treatment with Ang-(1–7)/MasR agonists rescues cognitive impairment and decreases plasma NfL relative to HF-saline-treated animals in our VCID mouse model. Further, levels of NfL are significantly negatively correlated with cognitive function and with several brain cytokine concentrations. Based on these preclinical findings, we propose that circulating NfL might be a candidate for a prognostic biomarker for VCID and may also serve as a pharmacodynamic/response biomarker for therapeutic target engagement.

## Introduction

Increases in brain and systemic inflammation, and decreased brain blood flow, as seen during heart failure (HF) [1], are strongly correlated with the development of vascular contributions to cognitive impairment and dementia (VCID) and Alzheimer's disease-related dementias (ADRD) [2, 3]. With an aging population and a strong link between cardiovascular and cerebrovascular disease and subsequent cognitive impairment and dementia, there is a growing need for safe and effective therapies to protect cognitive function in individuals at risk for VCID [4].

Angiotensin 1–7 (Ang-(1–7)) is a clinically safe peptide [5] that decreases inflammation and increases cerebral blood flow in our VCID preclinical model [6–8]. Through targeting Mas receptors (MasR), located on neurons, microglia, and vascular endothelial cells in the brain [9], Ang-(1–7) decreases brain inflammation and increases cerebral circulation [9]. MasRs that regulate vasodilation are highly expressed in the hippocampus and perirhinal cortex, which makes them an ideal target for treating brain disease related to hypoxia and inflammation that impair memory, such as VCID and ADRD [6, 7]. Our collaborative team has developed and optimized a novel, synthetic Ang-(1–7)-derived glycopeptide, PNA5, that selectively activates MasR and has improved bioavailability and brain penetration compared to the native Ang-(1–7) peptide. We have previously shown that PNA5 targets MasR, reverses cognitive impairment in our preclinical VCID model, and inhibits circulating and brain inflammatory cytokine production [7].

Neurofilament light protein (NfL) is an intermediate filament protein that is a component of the cytoskeleton of neurons and is abundantly expressed in axons [10, 11]. NfL levels are increased in the brain following axonal damage and neurodegeneration [10, 11] and increases in serum NfL correlate with cerebrospinal fluid (CSF) levels and increased cognitive impairment [12]. NfL has been found to be elevated in subjects suffering from multiple sclerosis [12, 13], as well as traumatic brain injury [14, 15], hypoxic brain injury [16], and cardiac disease and related surgeries [17].

In the present study, we hypothesized that (1) treatment with Ang-(1–7)/MasR agonists will rescue cognitive impairment and decrease VCID-induced NfL levels as compared to HF-saline treated mice, and (2) NfL levels will correlate with measures of both cognitive function and VCID-induced increases in brain cytokines. Our results indicate that circulating NfL might serve as a possible prognostic biomarker of cognitive impairment in VCID. Further, NfL might be a candidate biomarker to identify target engagement following treatment with Ang-(1–7)/MasR agonists in VCID.

## Methods

### Animal

Three-month-old (~ 25–30 g) C57BL/6 male mice (Jackson Laboratories, Bar Harbor, Maine) were ordered and housed 3 per cage in a temperature and humidity-controlled facility on a 12 h light/dark cycle. Mice are acclimated upon receival for a week before undergoing MI. Water and chow were available ab libitum for the duration of the experiments. Experiments were performed with adherence to guidelines approved by the Institutional Animal Care and Use Committee at the University of Arizona and in accordance with the National Institutes of Health Guidelines for the Care and Use of Laboratory Animals. The number of animals needed per experimental group was determined using a G*Power analysis.

### VCID animal model

Mice were weighed prior to surgery and anesthetized with 2.5% isoflurane in a mixture of air and O_2_. Left coronary artery (LCA) ligation was used to induce myocardial infarction (MI) using the procedure described in Gao et al. [18]. In short, a left-sided thoracotomy was performed at the fourth intercostal space and the LCA was sutured to induce a permanent ligation and MI. Occlusion of the LCA was confirmed by observing myocardial blanching of the left ventricular anterior wall. Control mice underwent sham surgeries, undergoing the same procedure except for the permanent LCA ligation.

### Experimental protocol

**Fig. 1:**
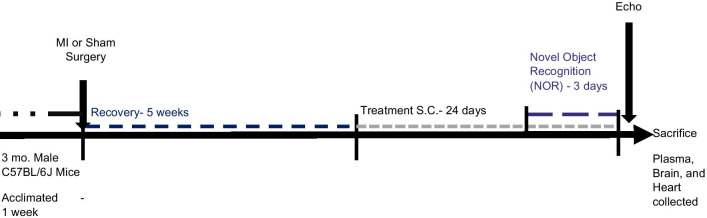
Experiment timeline. Three-month-old male mice were acclimated for one week before MI or Control surgeries. 5 weeks following recovery, mice were treated with Saline, Ang-(1–7) or PNA5 at 2 concentrations (50 and 500 ug/kg) for 24 days. During the last 3 days of treatment mice underwent Novel Object Recognition testing. Following Novel Object Recognition testing, mice underwent echocardiographs before killing. At killing, the plasma, brain, and heart were collected. *MI* myocardial infarct, *S.C*. subcutaneous injection

Mice were assigned randomly to the following treatment groups: Control-Saline (sham MI surgery, treated with saline), HF-treated (HF-PNA5, or HF-Ang-(1–7) with either 50 µg/kg/day, or 500 µg/kg/day), or HF-saline. Mice recovered for 5 weeks following MI before receiving 24 days of daily subcutaneous drug treatment. Within the last 3 days of injections, animals underwent Novel Object Recognition (NOR) testing (Fig. 1). Animal treatment groups were as listed: heart failure (HF)-saline treated n = 15, (HF-Saline), Sham surgery-saline treated n = 15 (Control-Saline), HF-Ang-(1–7) at 500 µg/ kilogram body weight/ day for 24 days (HF-A500) *n* = 15, HF-Ang-(1–7) at 50 µg/kg body weight/ day for 24 days (HF-A50) *n* = 15, HF-PNA5 at 500 µg/ kilogram body weight / day (HF-P500) *n* = 15, HF-PNA5 at 50 µg/ kilogram body weight/day (HF-P50) n = 15. Following MI, up to 7 mice died within a group resulting in varying number of mice per group that were available to undergo treatment, NOR testing, and provide serum samples.

### Echocardiography

Transthoracic, high-resolution ultrasound was performed the day of, or up to 2 days prior to, tissue harvesting using a Vevo 2100 High-Resolution Imaging System (Visual Sonics, Toronto, ON, Canada) and a 25-MHz transducer. Mouse chest hair was removed using a chemical depilatory. Echocardiographs were performed on anesthetized mice under 2% isoflurane and an O_2_/air mixture. Echocardiographic images were taken in B mode from the parasternal angle both on long and short-axis view at three levels starting at the apex, mid-ventricle, and top of the ventricle. The thickness of the left ventricular wall, cardiac chamber dimensions, interventricular septum, left ventricular posterior wall thickness, and left ventricular internal dimension were measured using Vevo 2100® analytic software (Visual Sonics, Toronto, ON, Canada). Only MI animals showing evidence of a significant MI, defined by significant wall motion abnormalities, and reduced contractile function, were included in the study.

### Preparation of Ang-(1–7) and the glycosylated Ang1-7, PNA5 peptides

To prepare the injections, solid peptides were dissolved in double distilled water and stored in a -20° C freezer in aliquots of 1.5 mL at a stock concentration of 1 mg/mL. The stock drugs were diluted daily in sterile saline to make the following treatment concentrations: A50 50 μg/kg (1.5 mg Ang-(1–7) /300 mL saline), P50 50 μg/kg (1.5 mg PNA5/300 mL saline), A500 500 μg/kg (15 mg Ang-(1–7)/300 mL saline), P500 500 μg/kg (15 mg PNA5/300 mL saline). Treatments were stored in a 4 °C refrigerator in prefilled syringes (peptide solutions are stable up to 28 days when stored at 4 °C).

### Subcutaneous injections

Daily treatment was delivered subcutaneously either at the scruff or inguinal area for 24 days using a 15-gauge needle. All prepared drugs were kept in the 4 °C refrigerator before use and transported on ice before injection. All procedures were performed within the housing facility to minimize stress.

### Novel Object Recognition (NOR) test

Mouse cognition was evaluated using the Novel Object Recognition test. For this test, the arena was an evenly illuminated chamber (12 × 12×12 cm) placed in a light and sound-controlled room. The walls of the arena were white, and the floor had a grid to ensure that the placement of the objects remained the same between phases of the test. Exploratory behavior was measured using a digital camera and evaluated by calculating the time spent exploring each object. Orientation of the nose toward an object at a distance of ∼2 cm or less was considered exploratory behavior [19]. Other behaviors including rearing on the object and resting against the object were not considered exploratory. Importantly, we have demonstrated that exploration is similar in both Control-Saline and HF-Saline treated mice, indicating that MI does not have a significant impact on mouse movement after 8 weeks of HF [6, 7]. Individuals scoring mouse exploration were blinded to treatment group. Three sets of distinct objects were used, variable in size, color, and shape, and made of either plastic, glass, or wood.

For the habituation phase of the test, mice were habituated to an empty testing arena for 10 min each day for 2 days. For the learning phase of the test, two identical objects were placed in the testing arena and the mouse was allowed to explore the objects for 6 min. After the 6 min of exploration, mice were returned to their home cage for 2 h. For the memory testing phase, one of the objects was replaced with a novel object and the mice were returned to the testing arena and allowed to explore the objects for 2 min. In between each phase, the testing boxes were cleaned with 70% ETOH to prevent the influence of olfactory cues. *Analysis:* Recognition memory was scored using a discriminatory ratio (discrimination ratio) calculated by: discrimination ratio = the time spent exploring the novel object (t_novel_) minus the time spent exploring the familiar object (t_familiar_) divided by the total exploration time:

1 $${\text{Discrimination ratio}} = \, \left( {t_{{{\text{novel}}}} - \, t_{{{\text{familiar}}}} } \right)/ \, \left( {\text{total exploration time}} \right).$$

A positive discrimination ratio score indicates that the mouse spent more time at the novel object than the familiar object, while a negative score indicates that the mouse spent more time with the familiar object than the novel object. A zero discrimination ratio score indicates a null preference. Typically, in healthy states, mice will spend a greater amount of time with the novel object than the familiar object. Discrimination ratios between groups were analyzed using ANOVA, followed by Dunnett’s post with significance set at *p* < 0.05.

### Measurement of neurofilament light (NfL)

Upon killing, blood was collected via heparinized syringe from the neck following decapitation. Collected blood was centrifuged and the plasma stored at -80 ͦ C before shipment to PBL Assay Science for measurement of NfL using a Quanterix-Simoa assay (NF-LIGHT® 103186, PBL, Neurofilament-Light Advantage Assay Kit).

### Brain lysis

Mice were anesthetized with isoflurane and rapidly decapitated. Brains were separated from the skull and quickly cut into two hemispheres. The halves of the brain were then rapidly frozen in corning tubes in liquid nitrogen and stored at -80° C. One half of each brain was lysed using 1 mL RIPA buffer (1% Triton X-100, 0.1% SDS, 1 × PBS) with protease (protease inhibitor cocktail 100x [Sigma P8340]) and phosphatase inhibitors (phosphatase inhibitor cocktail 100x [Sigma P5726]). Tissue was then sonicated using an ultrasonic cell disruptor at 30% 40% amplitude for 1 min. Samples were returned to ice in between sonication to prevent the tissue from warming. Samples were then centrifuged at 4 °C or 15 min at 13,000 × *g*. The supernatant was collected and stored at − 80 °C. Total protein was measured in the range of 5-10 mg/mL. Brain lysates were used for multiplex immunoassays.

### Cytokine measurement

Cytokine concentrations were measured in the brain lysates and plasma samples by multiplex immunoassay using a MAGPIX Multiplexing Instrument and accompanying Multiplex Analyst software (MILLIPLEX MAP Mouse Cytokine/Chemokine Magnetic Bead Panel-Immunology Multiplex Assay; MCYTOMAG-70 K; Millipore Sigma, Burlington, Massachusetts). Simultaneous measurements of granulocyte colony-stimulating factor (GCSF), granulocyte–macrophage colony-stimulating factor, interferon, interleukin (IL)-1a, IL-1B, IL-2, IL-4, IL-5, IL-6, IL-7, IL-9, IL-10, IL-12 (p40), IL-12(p70), IL-13, IL-15, IL-17, interferon–induced protein 10, chemokine(C-X-C motif) (CXC) ligand 1 (KC), chemokine (C–C motif) ligand(CCL) 2 (monocyte chemoattractant protein-1), CCL3 [macrophage inflammatory protein (MIP) 1a], CCL4 (MIP1b), CXC ligand 2 (MIP2),CCL5 [RANTES (regulated on activation normal T cell expressed and secreted)], and tumor necrosis factor-alpha (TNF-α) were captured. All samples were run in duplicate, and the results were analyzed using ANOVA and relevant post using GraphPad Prism9. To create a composite cytokine score, we calculated the *z*-scores for IL-2, IL-13, and IL-17 brain cytokines. Z-scores were then added to create a composite cytokine score. The *z-*score was calculated for each cytokine using the following equation:

2 $${\text{z score }} = \, \left( {{\text{individual value}} - {\text{mean value}}} \right)/{\text{SD}}{.}$$

### Heart tissue fixation

Mice were anesthetized with isoflurane and rapidly decapitated. The heart was exposed by opening the chest cavity and excised. The heart, while still beating, was then washed in ice cold PBS, dried, and weighed. Hearts were then cut longitudinally, exposing the interventricular septum and both ventricle chambers, and immersed in 10% formalin overnight at 4 °C. Hearts were then dehydrated in methanol with at least two methanol changes before being cleared with xylene for 45 min. Hearts were then wax permeabilized and paraffin embedded.

### Histochemistry

Paraffin-embedded hearts were sectioned on a microtome to a thickness of 5-microns and mounted onto polarized slides. They were then stained with hematoxylin and eosin to visualize heart morphology and cellular infiltrates.

### Statistical analysis

Data were analyzed using GraphPad Prism 9.0. Values are expressed as mean ± SE. The Shapiro–Wilk *W*-test was used for determining normal distribution of the data. Differences between multiple groups were analyzed with ANOVA followed by appropriate post hoc group comparisons where indicated. Comparison of NfL levels between the Control-Saline and HF-Saline groups were analyzed with an unpaired t-test. Statistical significance was set at *p* value < 0.05. Association between NfL levels and discrimination ratio scores and the brain cytokines were analyzed using Pearson correlation. Statistical significance was set at *p* value < 0.05. The discrimination ratio vs NfL linear regression and brain cytokine vs NfL linear regression was used to generate a best-fit line.

## Results

### HF in VCID model is confirmed through loss of ventricular function

**Fig. 2:**
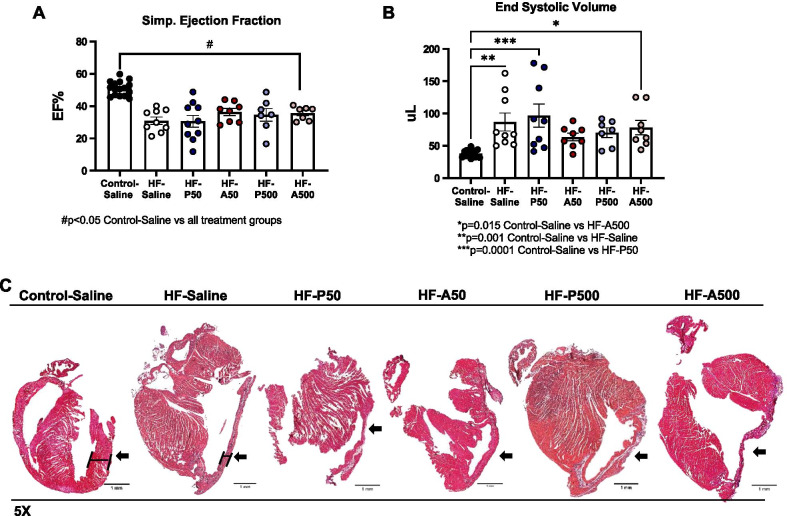
Echocardiograms and histology to confirm myocardial infarctions in the heart failure model.** A** Simpson Ejection Fraction was calculated using V-mode left ventricle calculations treatment groups: heart failure (HF)-A50 (Ang-(1–7)50 ug/kg), HF-P50 (PNA5 50ug/kg), HF-A500 (Ang-(1–7) 500 ug/kg), HF-P500 (PNA5 500 ug/kg), HF-Saline, Control-Saline. Significant difference between Control-Saline and the following treatment groups are as listed: HF-Saline, HF-A50 (Ang-(1–7)50 ug/kg), HF-P50 (PNA5 50 ug/kg), HF-A500 (Ang-(1–7)500 ug/kg), HF-P500 (PNA5 500 ug/kg), *P* < 0.05 ANOVA, Dunnett’s post. **B** Simpson End Systolic Volume was calculated using V-mode left ventricle calculation. Significant difference between Control-Saline and the following treatment groups are as listed: HF-Saline *p* = 0.001, HF-P50 (PNA5 50 ug/kg) *p* = 0.0001, and HF-A500 (Ang-(1–7)500 ug/kg) *p* = 0.015, ANOVA, Dunnett’s post. **C** Examples of hematoxylin and eosin staining of paraffin embedded formalin fixed hearts demonstrates differences in wall morphology between Control animals and HF-saline, PNA5, and Ang-(1–7) treatment mice. Left ventricle wall is indicated with black arrow. Images were taken at 5 × magnification

Our previous studies demonstrated that there is a significant deficit in cardiac function by 4 weeks post-MI [6, 7]. Accordingly, echocardiography was performed at 8 weeks post-MI using the Simpson’s method, the most used approach in clinical practice. As illustrated in Fig. 2A, at 8 weeks post-MI, ejection fractions (EF) were decreased in HF-Saline and Ang-(1–7) and PNA5-treated mice compared to Control-Saline-treated mice (Control-Saline, mean 50.98,± SE 1.12 *n* = 17 vs HF-Saline, mean 30.99± SE 2.26, *n* = 9, HF-A500, mean 35.56± SE 1.60, n = 7, HF-P500, 34.54 ± SE 3.86, n = 7, HF-P50, mean, 30.57± SE 3.66, *n* = 10, HF-A50 36.30± SE 2.18, *n* = 8; respectively, p ≤ 0.0002 ANOVA, Dunnett’s post). Further, at 8 week post-MI, end systolic volume (ESV) were significantly increased in all groups HF-Saline, as compared to Control-Saline treated mice (Control-Saline, mean 37.78± SE 1.30 *n* = 17 vs HF-VCID, mean 86.97± SE 13.90, *n* = 9, *p* = 0.0013, HF-A500, mean 78.29± SE 11.02, *n* = 8, *p* = 0.0149, HF-P50, 96.79± SE 17.87, *n* = 9, *p* = 0.0001; ANOVA, Dunnett’s post) (Fig. 2B). As observed in dilated hearts, our VCID mouse model has a thinned left ventricular wall [20]. Figure 2C is an illustrative example of left ventricular wall thinning in HF-Saline, PNA5, and Ang-(1–7) treatment treated mice compared to Control-Saline treated mice (indicated by black arrow).

### Ang-(1–7)/MasR activation rescues cognitive impairment in VCID mice

**Fig. 3:**
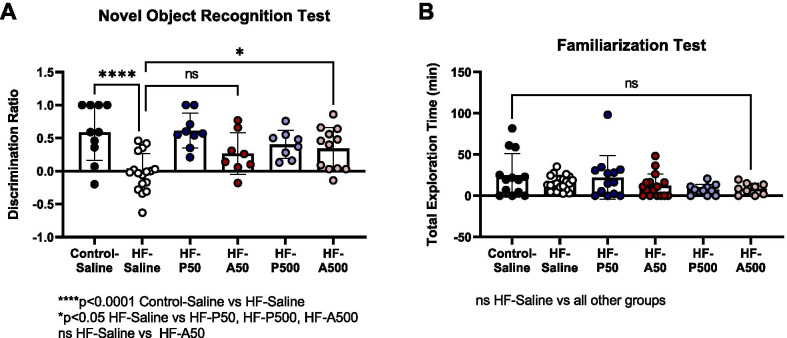
Ang-(1–7)/MasR agonists rescue cognitive impairment in a VCID mice.** A** Discrimination ratio = (Time spent at new object- Time spent at familiar object)/ total exploration time. Heart failure (HF) mice were treated with saline PNA5 or Ang-(1–7) at two different concentrations of either 50ug/kg, (P50, A50) or 500ug/kg (P500, A500). ****p < 0.001 Control-Saline vs HF-Saline. *p < 0.05 HF-Saline vs HF-P50, HF-P500, HF-A500. Data are represented with mean and ± SE. Difference in groups were tested by ANOVA, Dunnett’s post, significance *p* < 0.05. **B** No significant differences are noted in total exploration time in the familiarization test for all treatment groups. Data are represented with mean and ± SE. Significancy were tested by ANOVA

As illustrated in Fig. 3A, treatment with Ang-(1–7)/MasR agonists rescues cognitive impairment in our mouse model of VCID. Cognitive function was measured using the NOR test following 24 days of treatment with either 50 or 500 µg/kg/day of either Ang-(1–7) or PNA5. Cognitive function was quantified using the Discrimination ratio score, with a negative, or zero Discrimination ratio indicating impaired cognitive function. HF mice treated with saline had an average negative Discrimination ratio of − 0.026 ± SE 0.07 that was significantly lower than Control-Saline treated mice (mean 0.590± SE 0.13, *p* < 0.001, ANOVA, Dunnett’s post). This indicates, as previously described, that in our model of VCID, HF results in a decrease in cognitive function [6, 7].

Compared to HF-Saline animals, both treatment with PNA5 and Ang-(1–7) rescued the cognitive function in the VCID model. VCID mice treated with PNA5 at 50 or 500 µg/kg/day resulted in a discrimination ratio of 0.61 ± SE 0.08, *n* = 9, and 0.40± SE 0.08, *n* = 8, respectively (HF-P50 *p* < 0.0001, HF-P500, *p* = 0.01; ANOVA, Dunnett’s post). Likewise, treatment with Ang-(1–7) at 500 µg/kg/day also rescued HF-induced cognitive impairment mice (HF-A500, mean 0.34 ± SE 0.09, *n* = 12, *p* = 0.013; ANOVA, Dunnett’s post).

We have previously demonstrated that VCID mice do not demonstrate altered levels of activity or anxiety compared to control mice during the NOR test [6, 7]. Consistent with our previous publications, Fig. 3B demonstrates that our mouse model of VCID shows no significant difference in total exploration time during the familiarization phase in all groups. These results indicate that in comparison to Control-Saline-treated mice, VCID mice do not demonstrate altered levels of activity or anxiety (Control-Saline, mean 24.80 ± SE 7.32 *n* = 13, HF-Saline, mean 15.61 ± SE 1.91, *n* = 21, HF-P50, mean 22.32± SE 7.34, *n* = 13, HF-A50, 12.26 ± SE 3.59, *n* = 16, HF-P500, mean, 7.36± SE 1.93, *n* = 11, HF-A500 8.47± SE 2.34, *n* = 9; respectively, *p* > 0.05, not significant ANOVA).

### NfL plasma levels in VCID mice and effects of Ang-(1–7)/Mas receptor activation

**Fig. 4:**
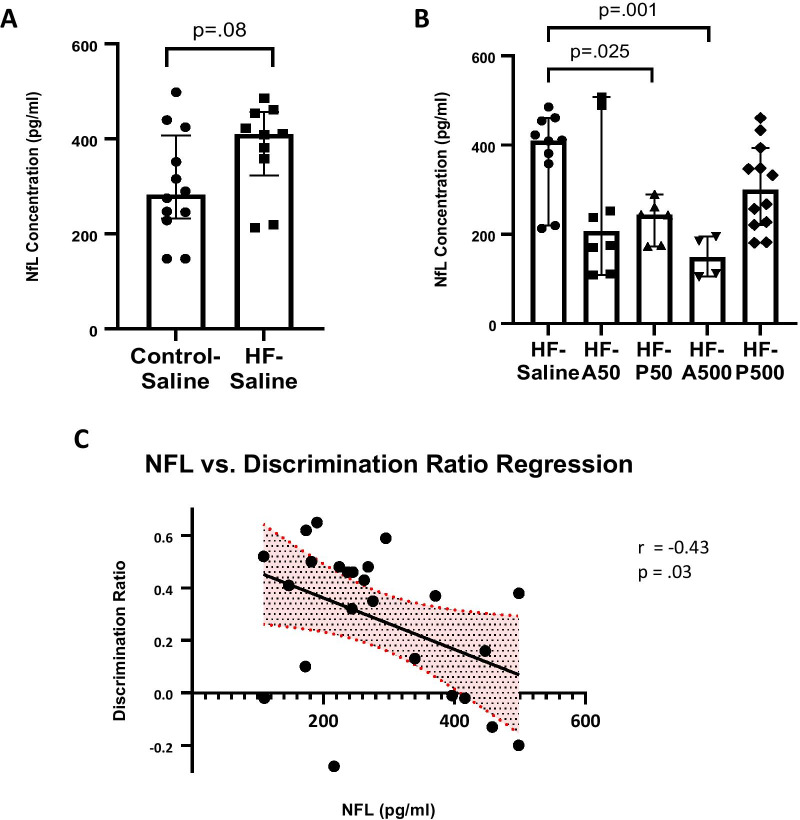
Treatment with Ang-(1–7)/Mas R agonists decreases plasma NfL levels. **A** Data are represented with mean and ± SE. Compares NfL levels between control saline and HF-saline mice, p = 0.08, Welch’s *t*-test. This difference did not reach significance. **B** PNA5 at 50ug/kg and Ang-(1–7) at 50 ug/kg, and 500 ug/kg significantly reduced NfL plasma concentration in VCID mice compared to HF-saline treated mice, Kruskal–Wallis ANOVA, Dunn’s post, *p* < 0.05. **C** NfL plasma levels significantly negatively correlated with Discrimination ratios. Correlation R values were produced using Pearson’s correlation analysis and the fit line was determined using simple linear regression

Levels of NfL were higher in the HF-Saline-treated mice relative to levels in the Control-Saline mice. The mean NfL level in the HF-VCID mice was 381.6 ± SE 30.0, (*n* = 10) as compared to Control-Saline-treated mice at 301.0 ± SE 32.0, (n = 12). Student’s t-test resulted in *p* = 0.08 and did not reach significance (Fig. 4A).

As illustrated in Fig. 4B, NfL concentrations were significantly lower following treatment with either A50, P50, or A500 as compared to HF-Saline-treated mice (HF-saline, mean 381.6 ± SE 32.0, (*n* = 12), HF-A50, mean 256.7 ± SE 55.8, (*n* = 8), *p* = 0.025; HF-P50 mean 231.1 ± SE 19.3, (*n* = 6), *p* = 0.025; HF-A500 mean 149.8 ± SE 23.6, (*n* = 4), *p* = 0.001; Kruskal–Wallis ANOVA, Dunn’s post).

To assess the relationship between cognitive impairment observed in our VCID model and circulating NfL, NfL levels were correlated to the discrimination ratio score of all mouse groups (Fig. 4C). Plasma NfL was significantly negatively correlated with the discrimination ratio score (Pearson’s *r* = − 0.43, *p* = 0.03), demonstrating that cognitive abilities decrease in this model as neuronal injury markers increase.

### Treatment with Ang1-7/MasR agonists affects levels of both brain and circulating cytokines

**Fig. 5:**
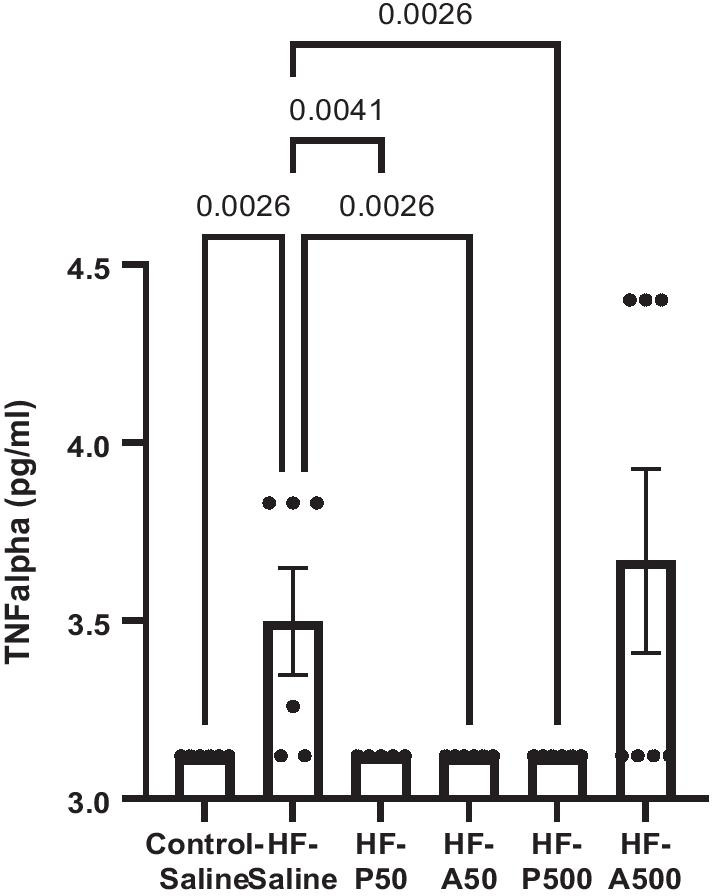
Increased TNFα in VCID models are inhibited by PNA5 and Ang-(1–7). Plasma TNFα of Control-Saline, HF-Saline, HF-A50 (Ang-(1–7)50 ug/kg), HF-P50 (PNA5 50ug/kg), HF-A500 (Ang-(1–7)500ug/kg), HF-P500 (PNA5 500ug/kg), were measured via MILLIPLEX MAP Mouse High Sensitivity Multiplex Immunoassay. Significance were observed between HF-Saline vs Control-Saline treated mice (3.50± SE 0.15, *n* = 6 vs mean 3.12, ± SE 0.01, *n* = 6, respectively, *p* = 0.003;), PNA5, and Ang-(1–7) (HF-P50 mean 3.12 pg/mL± SE 0.00, *n* = 5 *p* = 0.004; HF-A50 mean 3.12 pg/mL± SE 0.00, *n* = 6, *p* = 0.003; HF-P500 mean 3.12 pg/mL± SE 0.00, *n* = 6, *p* = 0.003; ANOVA, Dunnett’s post; values that were at or below assay threshold of 3.12 pg/mL were represented as 3.12 pg/mL)

Using the MILLIPLEX MAP Mouse High Sensitivity Multiplex Immunoassay, we measured plasma and brain inflammatory profiles after PNA5 and Ang-(1–7) treatments at both the 50 and 500 µg/kilograms/day doses. Among 25 cytokines measured in the plasma, only TNFα exhibited a significant difference between HF-Saline and Control-Saline-treated mice (3.50± SE 0.15, n = 6 vs means 3.12± SE 0.01, n = 6, respectively, p = 0.003; ANOVA, Dunnett’s post hoc). PNA5 and Ang-(1–7) inhibited this HF-induced increase in TNFα as compared to HF-saline groups (HF-P50 mean 3.12 pg/mL± SE 0.00, *n* = 5 *p* = 0.004; HF-A50 mean 3.12 pg/mL± SE 0.00, *n* = 6, *p* = 0.003; HF-P500 mean 3.12 pg/mL± SE 0.00, *n* = 6, *p* = 0.003, ANOVA, Dunnett’s post; values that were at or below assay threshold of 3.12 pg/mL were represented as 3.12 pg/mL) (Fig. 5).

**Fig. 6:**
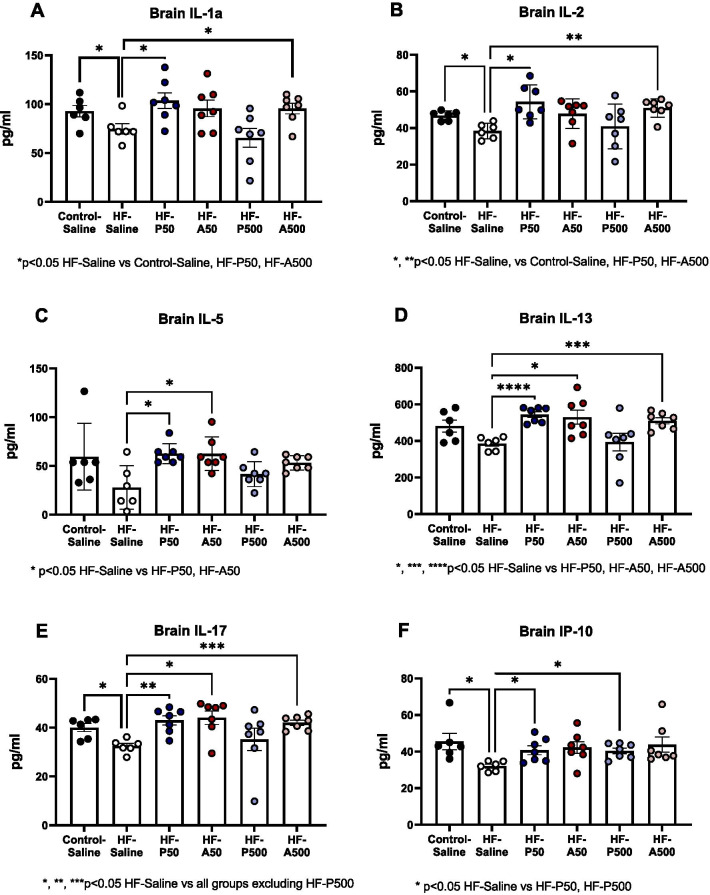
Effect of treatment with Ang-(1–7)/MasR agonists on brain cytokine levels. Whole brain lysate cytokine levels were measured using MILLIPLEX MAP Mouse High Sensitivity Multiplex Immunoassay. **A**–**F** HF-Saline treated VCID mice had significantly lower IL-1α, IL-2, IL-17, and IP-10 levels in comparison to Control-Saline treated mice. PNA5 and Ang-(1–7) significantly increased cytokines levels as compared to HF-saline treated animals. Data are represented with mean and ± SE. Differences in groups were tested by ANOVA, Dunnett’s post hoc, significance set a *p* < 0.05

Cytokine levels from brain lysates are summarized in Table 1 and illustrated in Fig. 6. Of the 25 cytokines tested treatments with P50 significantly increased levels of putative-neuroprotective cytokines IL-1α, IL-2, IL-5, IL-13, IL-17, and IP-10, compared to HF-Saline treated mice, ANOVA, Dunnett’s post hoc, *p* < 0.05 (Fig. 6A–F). Similarly, treatments with A50 or A500 also significantly increased levels of IL-1α, IL-2, IL-5, IL-13, and IL-17 in comparison to HF-Saline-treated mice, ANOVA, Dunnett’s post, p < 0.05. Lastly, IL-1α, IL-2, IL-17, and IP-10 were significantly decreased in HF-Saline-treated mice in comparison to Control-Saline treated mice (Table 1).

**Table 1:** Table demonstrating cytokine and chemokine levels of whole brain lysate

	Cytokine and chemokine, relative expression (pg/ml)						*p* values				
Cytokines	Control-Saline (*n*=5-6)	HF-Saline (*n*=6)	HF-A50 (*n*=6-7)	HF-P50 (*n*=6-7)	HF-A500 (*n*=7)	HF-P500 (*n*=7)	Control-Saline vs. HF-Saline	HF-Saline vs. HF-P50	HF-Saline vs. HF-A50	HF-Saline vs. HF-P500	HF-Saline vs. HF-A500
IL-1α	92.8 ±5.9	74.7 ±5.4	95.6 ±8.4	103.7 ± 8.1	95.6 ± 5.5	65.4 ±9.7	0.048*	0.027*	ns	ns	0.040*
IL-2	46.8 ±1.1	38.4 ±1.7	47.8 ±3.0	54.3 ± 3.5	50.9 ± 1.9	40.9 ±4.6	0.015*	0.013*	ns	ns	0.002*
IL-5	59.5 ±14.0	28.0 ±9.2	62.6 ±3.9	62.6 ±3.9	53.2 ±2.7	41.7 ±4.9	ns	0.020*	0.026*	ns	ns
IL-13	481.1 ±33.4	385.9 ±14.8	529.9 ±38.6	543.8 ±15.6	510.0 ±17.3	394.0 ±47.6	ns	<0.0001*	0.036*	ns	0.001*
IL-17	40.0 ±1.7	32.4 ±1.1	44.1 ± 2.8	43.0 ±1.9	42.0 ±1.1	35.2 ±4.6	0.021*	0.004*	0.020*	ns	0.0003*
IL-10	45.5 ±4.4	32.1 ±1.1	42.2 ±3.2	40.8 ±2.5	43.8 ±4.2	40.4 ±1.5	0.049*	0.023*	ns	0.005*	ns

IL-1α, IL-2, IL-5, IL-13, IL-17, and IP-10, mean, standard error and number of samples are provided for the following treatment groups: Control-Saline, HF-Saline, HF-A50 (Ang-(1–7), Ang-(1–7)50 ug/kg), HF-P50 (PNA5 50 ug/kg), HF-A500 (Ang-(1–7)500 ug/kg), and HF-P500 (PNA5 500ug/kg). Significant difference between treatment groups and HF-Saline are provided, *Indicates significance, *p* < 0.05, (ANOVA, Dunnett’s post hoc), *ns* not significant)

**Fig. 7:**
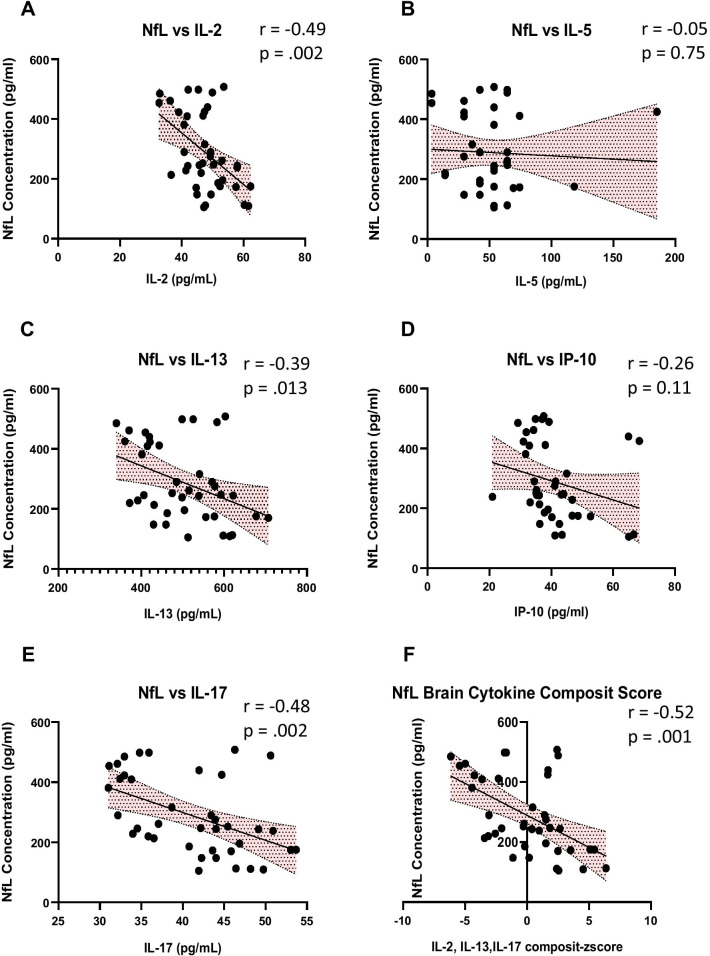
Relationship between brain cytokine levels and NfL levels.** A**–**E** The relationship between brain cytokines IL-2, IL-5, IL-13, IL-17, IP-10, and plasma NfL levels were analyzed using Pearson correlation. **F** Brain cytokine composite score of IL-2, IL-13, IL-17 were significantly negatively correlated with NfL plasma concentrations

**Fig. 8:**
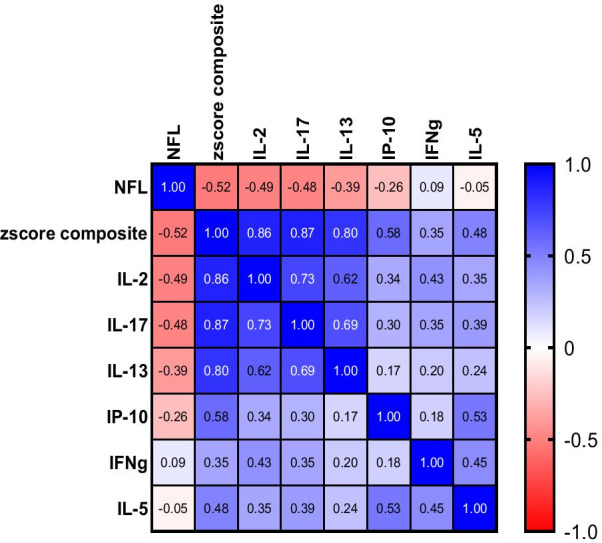
Pearson’s correlation matrix of the relationship between plasma NfL and brain cytokine levels. The relationship between the brain cytokines IL-2, IL-5, IL-13, IL-17, IP-10, the composite cytokine z-score and plasma NfL levels were analyzed using Pearson correlation. Blue demonstrates positive correlation and red represents negative correlation. Values closer to 1 or -1 indicate more closely correlated variables

The relationship between levels brain cytokines and NfL was analyzed using Pearson correlation of NfL concentrations and brain cytokines (Table 2, Fig. 7). IL-2, IL-13, IL-17, and the combined cytokine composite score were significantly negatively correlated with NfL plasma concentrations. Figure 8 is a Pearson’s correlation matrix illustrating the correlation coefficients between NfL and those brain cytokines described in Table 2. These data show that (1) NfL is most strongly negatively correlated with the cytokine-composite score and, (2) the levels of IL-2, IL-17 and IL-13 are all strongly positively correlated with each other.

**Table 2:** NFL vs brain cytokine correlations

Cytokines	IL-2	IL-5	IL-13	IP-10	IL-17	Cytokine composite z-score
*r* value	− 0.49	− 0.05	− 0.39	− 0.26	− 0.48	− 0.52
*p* value	.002	0.74	.013	0.11	.002	.001

IL-2, IL-5, IL-13, IL-17, IP-10 and the composite cytokine z-score, Pearson’s correlation r values and affiliated *p* values following correlation analysis of NfL levels and cytokine levels in all animal groups are shown. *Indicates significance, *p* < 0.05, *ns* not significant

### Longitudinal comparisons of circulating inflammatory cytokine and chemokines

**Fig. 9:**
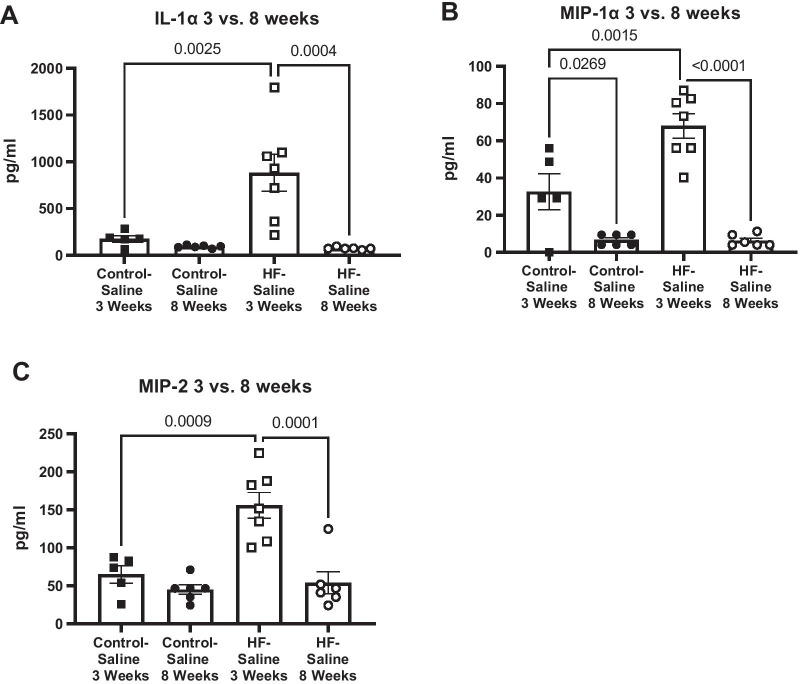
Longitudinal comparison of plasma cytokines in VCID mouse model.** A**–**C **Plasma inflammatory cytokine levels from mice with 3 weeks of HF, and 8 weeks of HF, in both Control-Saline, and HF-Saline treated mice were measured via MILLIPLEX MAP Mouse High Sensitivity Multiplex Immunoassay. At 3 weeks of HF, HF-Saline mice had significantly higher IL-1α, MIP-1α, and MIP-2 concentrations than age-matched Control-Saline treated mice. A significant decrease in cytokine concentrations was observed in HF-Saline-treated mice from 3 to 8 weeks of HF. Data are represented with mean and ± SE. Difference in groups were tested by ANOVA

HF is a progressive condition, worsening over time. To highlight the longitudinal impact of HF-induced inflammation, we compared plasma cytokines at 3 weeks post-MI, before the onset of HF, to 8 weeks post-MI, when HF has been established (Fig. 9). A significant decrease in IL-1α, MIP-1α, and MIP-2 plasma concentrations was observed in HF-Saline treated mice between the two time periods (Table 3). Unlike IL-1α, and MIP-2, MIP-1α demonstrated decreased levels 8 weeks post-MI compared to 3 weeks post-MI in both Control-Saline, and HF-Saline treated mice. (IL-1α 3 weeks HF mean 882.3 pg/mL ± SE 198.4, *n* = 7 vs 8 week HF mean 209.6 pg/mL± SE 55.7, n = 6, *p* = 0.0004; MIP-1α 3 week HF mean 67.9 pg/mL± SE 6.6, *n* = 7 vs 8 week HF mean 6.4 pg/mL± SE 1.3, *n* = 6, *p* < 0.0001; MIP-2 3 weeks HF mean 155.9 pg/mL± SE 17.0, *n* = 7 vs 8 weeks HF mean 54.1 pg/mL± SE 14.7, *n* = 6, *p* = 0.0001; ANOVA, Tukey’s post).

**Table 3:** Longitudinal values of HF-induced inflammation comparing plasma cytokines at 3 weeks post-MI to 8 weeks post-MI

	Cytokine and chemokine, relative expression				*p* values		
	3 weeks HF	3 weeks HF	8 weeks HF	8 weeks HF	3 weeks Control-Saline vs. 3 weeks HF-Saline	3 weeks Control-Saline vs. 3 weeks HF-Saline	3 weeks HF-Saline vs. 8 weeks HF-Saline
Cytokines	Control-Saline (*n*=5)	HF-Saline (*n*=7)	Control-Saline (*n*=6)	HF-Saline (*n*=6)			
IL-1α	176.1 ± 34.8	882.3 ±198.4	92.8 ± 5.9	74.7 ±5.4	0.003*	0.964	0.0004*
MIP-1α	32.6 ±9.7	67.9 ±6.6	6.8 ± 1.2	6.4 ±1.3	0.002*	0.027*	<0.0001*
MIP-2	65.0 ±11.4	155.9 ±17.0	45.2 ±6.3	54.1 ±14.7	0.0009*	0.764	0.0001*

Mean, Standard error, and number of sample values of plasma IL-1α, MIP-1α, and MIP-2 from 3 and 8 weeks post-MI are represented for both treatment groups Control-Saline, and HF-Saline. *P* values of the following comparison are provided: 3 weeks post-MI Control-Saline and 3 weeks post-MI HF-Saline, 3 weeks post-MI Control-Saline and 8 weeks post-MI Control-Saline, and 3 weeks post-MI HF-Saline and 8 weeks post-MI HF-Saline. *Indicates significance, *p* < 0.05, (ANOVA, Tukey’s post), *ns* not significant

## Discussion

Vascular contributions to cognitive impairment and dementia (VCID) and Alzheimer’s disease-related dementias (ADRD) significantly contribute to the 47 million people world-wide who suffer with dementia. This number is estimated to increase to over 130 million people by 2050. A number of studies have shown that VCID and conversion to ADRD are strongly correlated with vascular disease, inflammation and decreased cerebral brain blood flow [21–28]. The relationship between vascular disease, cognitive function and progression to dementia and possible AD have been recently reviewed [4, 29, 30]. These authors successfully make the case for a close relationship between cardiovascular risk factors and risk for VCID and ADRD. Furthermore, conversion rates of VCID to dementia has been reported to be within 40–46% within 5 years of diagnosis of VCID [31, 32]. There is an urgent unmet medical need for therapeutics to prevent cognitive decline in individuals at risk for VCID.

In this study, we demonstrate that treatment of our VCID mice with Ang-(1–7)/MasR agonists reverse cognitive dysfunction and decrease circulating NfL as compared to HF-saline-treated animals. This change in NfL level is significantly negatively correlated with cognitive function. These early preclinical data suggest that NfL levels might serve as a prognostic to identify changes in cognitive function in VCID. In addition, NfL significantly correlated with treatment induced changes in brain cytokines further suggesting that NfL may potentially also serve as a pharmacodynamic/response biomarker for therapeutic target engagement and disease modification. Extensive studies on levels of NfL in humans with VCID are needed to confirm the utility and reliability of NfL as a biomarker for cognitive impairment in individuals at risk for VCID.

### NfL as a biomarker in neurodegenerative diseases

NfL is released into the CSF and blood upon axonal damage [10]. Increases in CSF and blood concentrations of NfL have been found in multiple neurodegenerative diseases [33] as well as traumatic brain injury [14], hypoxic brain injury [16], and cardiac disease [17]. Blood NfL levels have been proposed as a biomarker of cognitive decline in Alzheimer’s disease (AD) and Parkinson’s disease (PD) patients [33, 34. It has been demonstrated that patients experiencing mild cognitive impairment and dementia stages of AD have increased blood and CSF NfL levels [35], from these results it has been previously proposed that NfL may be able to help predict the progression of AD dementia in patients [33, 34].

Additionally, it has been demonstrated that CSF NfL levels are increased in patients with vascular dementia [36, 37] and that increased NfL levels are correlated to lower MMSE scores [37]. Further, it has been shown that patients with vascular dementia demonstrate a correlation between increased NfL serum levels and decreased cognitive impairment [38]. Studies including CADASIL (a genetic disorder affecting the small blood vessels in the brain), and small vessel disease (SVD) VCID patients also demonstrated increases of NfL correlated to worsened processing speed [39]. In the present study, we provide preclinical support for the hypothesis that NfL may be a prognostic biomarker, as described by the FDA BEST Biomarker Working Group [40] for the identification of cognitive impairment in individuals at risk for VCID.

In the present study we have also shown that treatment with Ang-(1–7)/MasR agonists can decrease NfL concentrations in our VCID model as compared to HF-saline treated animals and this decrease in NfL is inversely correlated to several pleiotropic cytokines found in the VCID mouse brain. The correlation of blood NfL levels with neurodegenerative treatment protocols has been demonstrated in previous human studies. In MS patients, studies have shown that blood levels of NfL correlate with MS related disability and can predict the longitudinal course of the disease as well as response to treatment [41–43].

### Systemic and brain inflammation

A number of different mechanisms have been suggested to contribute to VCID [44, 45]. Systemic inflammation has been linked to impaired cognitive function [46, 47]. Specifically circulating TNFα is increased in HF patients [48, 49] and has been demonstrated to contribute to pathologic cognitive changes [47]. In this study we observed that TNFα was significantly increased in the HF-Saline treated mice compared to the Control-Saline treated mice, thereby possibly contributing to systemic inflammation in our VCID mouse model. Importantly, treatment with Ang-(1–7)/MasR agonists in VCID mice reversed not only cognitive impairment, but also decreased NfL levels, and systemic TNFα concentrations. These data provide further evidence that NfL might serve as a biomarker to indicate target engagement of the disease-modifying treatment with our Ang-(1–7)/MasR agonists.

We have also begun to identify longitudinal changes in circulating inflammatory cytokine profiles in our VCID mouse model. We compared changes in systemic cytokines in animals with 3 weeks of HF to animals with 8 weeks of HF. Previously, we have identified inflammatory biomarkers associated with the early progression of VCID following the onset of HF (3 weeks following MI) [6]. IL-1α, MIP-1α, MIP-2, and GCSF in HF-Saline mice were significantly increased compared to the Control-Saline treated mice 3 weeks following MI [6]. These data reveal a longitudinal decrease in IL-1α, MIP-1α, and MIP-2 which provides insight into the inflammatory mechanism of VCID development.

Regarding brain cytokines, HF-Saline treated mice had decreased levels of IL-1α IL-2, IL-17, and IP-10 in comparison to Control-Saline treated mice. Treatment with P50 significantly increased IL-1α, IL-2, IL-5, IL-13, IL-17 and IP-10 levels, and treatments with Ang-(1–7) also significantly increased levels of IL-1α, IL-2, IL-5, IL-13, and IL-17 in comparison to HF-Saline treated mice.

IL-1α has been shown to have brain protective qualities in mouse stroke models and promotes neurorepair when mice are treated with delayed administration of IL-1α [50]. Other studies have demonstrated that IL-1α induces angiogenesis in brain endothelial cells in vitro [51]. Further it was observed that IL-1α levels increase within the brain in post-stroke animal models during angiogenic periods [51]. In the current study IL-1α was significantly lower in HF-Saline treated mice compared to Control-Saline treated mice, and that treatment of P50, or A500 increases IL-1α significantly in comparison to HF-Saline mice. Suggesting that treatment with Ang-(1–7)/MasR agonists may provide brain protection and neurorepair by increasing brain IL-1α levels.

IL-2 has also been shown to play a protective role in the brain. IL-2 has been shown to decrease amyloid plaque load, improve synaptic plasticity, and has been associated with memory recovery in AD mice [52]. We observed that IL-2 brain levels significantly decreased in our VCID model in comparison to controls. We further demonstrated that treatment with P50, or A500 significantly increased IL-2 levels in comparison to HF-Saline mice, and that IL-2 levels were significantly negatively correlated with NfL levels. These data suggest that one of the mechanisms by which Ang-(1–7)/MasR treatment protects cognitive function may include increasing IL-2.

IL-5 is a Th2 cytokine that can act as an immunosuppressor [53] and contributes to maintaining homogeneity of astrocyte activation states [54]. Elevated serum levels of IL-5 have been have been reported in both VCID patients and vascular encephalopathy (VE) [53]. It has been suggested that elevated levels of IL-5 in VCID and VE may contribute to disease reduction. In PC12 cells, treatments of IL-5 decreased neuronal cell apoptosis, and Aβ_25-35_ induced tau phosphorylation [54]. In the present study we show that treatments with P50, or A50 significantly increase brain IL-5 levels compared to HF-Saline mice. These data suggest that elevated IL-5 levels may contribute to the mechanism by which Ang-(1–7)/MasR treatment protects cognitive function.

IL-13 has been shown to play both a protective and injurious role in the brain. While IL-13 has been demonstrated to be neuroprotective by modulating microglia/macrophage responses in traumatic brain injury [55], other studies have shown that IL-13 can be detrimental to neuronal survival [56]. Here we found that treatment with P50, A50, and A500 significantly increase IL-13 levels in comparison to HF-Saline treated mice. The IL-13 levels were significantly negatively correlated to NfL plasma concentrations. These data indicate that IL-13 may play a neuroprotective role following treatment with Ang-(1–7)/MasR agonists in our mouse model of VCID.

IL-17 has also been shown to play both a protective and injurious role in the brain. Although IL-17 is typically linked to proinflammatory disease progression [57], it has also been shown to promote tissue repair via meningeal resident gamma delta T-cells and support long-term potentiation (LTP) and the plasticity of glutamatergic synapses during short-term learning [58]. We observed that HF-Saline treated mice had significantly lower levels of IL-17 than Control-Saline treated mice, and that treatments with P50, A50, or A500 normalized IL-17 brain concentrations. Further, levels of IL-17 were significantly negatively correlated with Nfl levels. These findings suggest that Ang-(1–7)/MasR agonists increase IL-17 levels in VCID mice, which may contribute to these peptides’ cognitive protective effects.

IP-10 (CXCL-10) has been demonstrated to increase in the pathogenesis of AD [59]. In contrast, studies with AD patients demonstrate an increased in CSF and plasma IP-10 levels were independently associated with NfL levels [60]. In our model of VCID, we observed IP-10 brain levels were significantly higher in both Control-Saline-treated mice and Ang-(1–7)/MasR agonist-treated HF mice as compared to HF-Saline mice.

In total, these data suggest that in our VCID model there is a decrease in the levels of multiple pleiotropic cytokines in HF-saline-treated animals. Treatment with Ang-(1–7)/MasR agonists mitigated cognitive impairment in this model while simultaneously increasing the levels of these pleotropic, and some putative neuroprotective cytokines, some of which are correlated to decreases blood NfL levels.

Finally, while extensive human studies are needed to support this premise, we propose that NfL may be a potential predictive biomarker to identify individuals who are at risk for VCID and pharmacodynamic biomarker to indicate target engagement following Ang-(1–7)/MasR therapy to protect cognitive function.

### Study limitations

One limitation of this study is the lack of data in female animals. We appreciate that effects of both HF and drug treatment may be different in female animals, and we have plans to fully investigate the effects of HF and Ang-(1–7)/MasR treatments in female mice in future studies. A second limitation of this study, is the lack of complete longitudinal data showing the change in circulating and brain cytokines over the course of development of the HF post-MI. While our results comparing 3-week to 8-week post-MI levels of cytokines in both plasma and brain suggest that the levels of these cytokines do change during disease progression a more extensive analysis of the time-course of these changes and the time-course of the effectiveness of treatment with Ang-(1–7)/MasR therapies will be needed to better understand the inflammatory mechanisms underlying the development of VCID.

## Abbreviations

Ang-(1–7): Angiotensin 1–7
A50: Ang-(1–7) at 50ug/kg
A500: Ang-(1–7) at 500ug/kg
ADRD: Alzheimer disease-related dementia
Echo: Echocardiograph
EF: Ejection fraction
ESV: End systolic volume
HF: Heart failure
NfL: Neurofilament light
MI: Myocardial infarct
P50: PNA5 at 50ug/kg
P500: PNA5 at 500ug/kg
ROS: Reactive oxidative species
SVD: Small vessel disease
VaD: Vascular dementia
VCID: Vascular contributions to cognitive impairment and dementia
VE: Vascular encephalopathy
